# A novel peroxidase from *Ziziphus jujuba* fruit: purification, thermodynamics and biochemical characterization properties

**DOI:** 10.1038/s41598-020-64599-9

**Published:** 2020-05-14

**Authors:** Mustafa Zeyadi, Yaaser Q. Almulaiky

**Affiliations:** 1 Department of Biochemistry, Faculty of Science, King Abdulaziz University, Jeddah, P. O. Box 80200, Jeddah, 21589 Saudi Arabia; 2 University of Jeddah, College of Sciences and Arts at Khulais, Department of Chemistry, Jeddah, Saudi Arabia; 3 Chemistry Department, Faculty of Applied Science, Taiz University, Taiz, Yemen

**Keywords:** Biochemistry, Isoenzymes

## Abstract

In this study, peroxidase from *Ziziphus jujuba* was purified using ion exchange, and gel filtration chromatography resulting in an 18.9-fold enhancement of activity with a recovery of 20%. The molecular weight of *Z. jujuba* peroxidase was 56 kDa, as estimated by Sephacryl S-200. The purity was evaluated by SDS, which showed a single prominent band. The optimal activity of the peroxidase was achieved at pH 7.5 and 50 °C. *Z. jujuba* peroxidase showed catalytic efficiency (Kcat/Km) values of 25 and 43 for guaiacol and H_2_O_2_, respectively. It was completely inactivated when incubated with β-mercaptoethanol for 15 min. Hg^2+^, Zn^2+^, Cd^2+^, and NaN3 (5 mM) were effective peroxidase inhibitors, whereas Cu^2+^ and Ca^2+^ enhanced the peroxidase activity. The activation energy (Ea) for substrate hydrolysis was 43.89 kJ mol^−1^, while the Z value and temperature quotient (Q_10_) were found to be 17.3 °C and 2, respectively. The half-life of the peroxidase was between 117.46 and 14.15 min. For denaturation of the peroxidase, the activation energy for irreversible inactivation Ea*(d) was 120.9 kJmol^−1^. Thermodynamic experiments suggested a non-spontaneous (∆G*d > 0) and endothermic reaction phase. Other thermodynamic parameters of the irreversible inactivation of the purified enzyme, such as ∆H* and ∆S*, were also studied. Based on these results, the purified peroxidase has a potential role in some industrial applications.

## Introduction

Peroxidase (E.C.1.11.1.7) belongs to the class of oxidoreductases, containing iron (III) protoporphyrin IX as the prosthetic group, usually present in plants and responsible for the process of brewing^1,2^. Class III plant peroxidase is a normal enzyme whose activity was already identified in 1855 and which was purified several decades later^3^. peroxidase is relatively stable at high temperatures, and its activity can be easily measured using simple chromogenic reactions. The oxidation of different phenolic and non-phenolic substrates that participate in the breakdown of H_2_O_2_, is catalysed by peroxidases, which are major antioxidant enzymes. Peroxidases play an important role in industrial applications, since peroxidases can catalyse the broad range of redox reactions in the presence of H_2_O_2_^4,5^. Peroxidase enzymes have various important roles not only in the biomedical industry (diagnosis kit development, organic, immunoassays, and polymer synthesis^6^, as well as in biosensor technology^7^) but also in the agriculture industry and its allied sectors^8^. These also have important roles in plant physiology processes, such as cell safety against oxidative stress, the formation of lignin and suberin, crosslinking of cell wall components, wound healing, protection against pathogens or insects^9^, and promote plant darkening^10^. Peroxidase causing the oxidation crosslinking of pentosan in the dough. Oxidative enzymes such as peroxidase are added in bread dough to avoid stickiness. The positive effect of peroxidases on the breading process is due to the cross-linking of feruloylated arabinoxylans in larger aggregates. These have a better ability to hold water and enable water transfer in the dough. Additionally, peroxidase can influence the gluten network either by cross-linking the gluten proteins or by adding arabinoxylans to gluten proteins^11^. Peroxidase enzymes are also reported for bioremediation of various phenolic dyes and decolorizers^12^. The catalytic reaction of peroxidase occurs in three stages. The initial stage involves peroxidase oxidation to create an unstable intermediate compound called Cpd I (1). In the second stage, a corresponding electron donor reduces Cpd I to Cpd II and to a free radical (2). Then the second substrate further lowers Cpd II to restore the enzyme’s resting state and a radical one (3)^13^.

1 $${\rm{Enz}}({\rm{Por}}-{{\rm{Fe}}}^{{\rm{III}}})+{{\rm{H}}}_{2}{{\rm{O}}}_{2}\to \textregistered {\rm{CPd}}\,{\rm{I}}({{\rm{Pro}}}^{\cdot +}-{{\rm{Fe}}}^{{\rm{IV}}}={\rm{O}})+{{\rm{H}}}_{2}{\rm{O}}$$

2 $${\rm{CPd}}\,{\rm{I}}({{\rm{Pro}}}^{\cdot +}-{{\rm{Fe}}}^{{\rm{IV}}}={\rm{O}})+{{\rm{AH}}}_{2}\to {\rm{CPd}}\,{\rm{II}}({\rm{Pro}}-{{\rm{Fe}}}^{{\rm{IV}}}-{\rm{OH}})+{{\rm{AH}}}^{\cdot }$$

3 $${\rm{CPd}}\,{\rm{II}}({\rm{Pro}}-{{\rm{Fe}}}^{{\rm{IV}}}-{\rm{OH}})+{{\rm{AH}}}_{2}\to \textregistered {\rm{Enz}}({\rm{Por}}-{{\rm{Fe}}}^{{\rm{III}}})+{{\rm{AH}}}^{\cdot }$$

*Ziziphus jujuba* generally known as annep in Saudi Arabia, belongs to the *Rhamnaceae* family, which consists of 550 species and 45 genera that are commonly distributed in the tropical and subtropical regions around the world^14^. *Ziziphus jujuba* is a hardy tree present in arid regions and unfavourable growth conditions include saline soil in a hot and arid environment^15^. The chemical composition of jujubes has been studied by several researchers^15,16^. Jujuba has been consumed since ancient times and is still a popular and influential fruit in human diets. The recent pharmacological and phytochemical results have shown that triterpenic acid, flavonoids and polysaccharides are the main active components within jujubes^17–20^. Jujuba polysaccharides are suggested to be the primary active components due to their haematopoietic, immunomodulatory and haematopeutic roles^21,22^. Additionally, due to their anticancer and anti-inflammatory properties, triterpenic acids were considered the active ingredients of jujubes^23,24^. Moreover, jujuboside B and betulinic acid could be the active components, as they display beneficial effects on the cardiovascular system^25,26^. Various studies have supported the biological activities of jujube, which is considered medicinal herb as well as a food. In Chinese medicinal theory, jujube was considered a herb that can relieve mental stress and can calm the state of mind. In clinical practices, jujube is taken alone or combined with other herbal medicines to treat forgetfulness and insomnia. Recent reviews have summarized the composition of the jujube as well as its health benefits^27,28^.

In the past, researchers have studied the characterization and purification of certain kinds of peroxidase enzymes, such as Arabian balsam peroxidase^29^, Kalipatti sapota peroxidase^30^, pearl millet grains peroxidase^31^, and green gram root peroxidase^32^. Nevertheless, to our full knowledge, there is no study on the purification and characterization of peroxidase from on *Z. jujuba* fruit.

This research was therefore aimed purifying and characterizing peroxidase from *Z. jujube* and to investigate the potential contribution of *Z. jujuba* peroxidase in the production and storage of fresh *Z. jujuba* in order to select an appropriate method for mitigating peroxidase activity. The negative effect of peroxidase is causing unhealthy fruit browning and vegetable off-flavors^33^. *Z. jujuba* is seasonal fruit, and easily perishable. Thus, this research was therefore aimed purifying and characterizing peroxidase from *Z. jujube* and to investigate the physical, biochemical and thermodynamic characteristics of the peroxidase enzyme so that the conditions can be regulated for mitigating peroxidase activity that causes unhealthy fruit browning and then increases the fruit storage time.

## Material and methods

### Materials

Jujube (*Z. jujuba*) fruit were collected from Al-Baha City, Saudi Arabia. DEAE-Sepharose, Sephacryl S-200, hydrogen peroxide, guaiacol and 4-aminoantipyrine were acquired from Sigma-Aldrich (St. Louis, MO, USA). All other chemicals used were of analytical grade.

### Purification of *Z. jujuba* peroxidase

#### Preparation of crude extract

Twenty grams of *Z. jujuba* skin were ground in a 20 mM Tris–HCl buffer at pH 7.2. This crude extract was filtered and centrifuged at 10,000 rpm for 10 min and the pellet was discarded

#### Ion exchange and gel filtration chromatography

A chromatographic column was packed with DEAE-Sepharose. It was equilibrated with 20 mM Tris–HCl buffer at pH 7.2. Then, the crude extract enzyme was loaded onto the column and washed with equilibrating buffer. Proteins were eluted with a stepwise gradient of 0.0–0.3 M NaCl in the same buffer. The fractions were collected and spectrophotometric absorption was measured at 280 nm. The peroxidase activity of the fractions showing 280 nm absorbance was measured at 470 nm. The peroxidase fractions with the highest activity were concentrated by lyophilization and loaded onto a Sephacryl S-200 column that had previously been equilibrated with the 20 mM Tris–HCl buffer at pH 7.2. A 30 ml h^−1^ flow was used to collect 3 ml fractions

### Protein determination

The Bradford method was used for measurement of the protein content^34^, using bovine serum albumin as a standard.

### Enzyme assay

peroxidase enzyme activity was determined according to Yuan and Jiang^35^. One millilitre of a reaction mixture containing 0.008 M hydrogen peroxide, 0.04 M guaiacol, 0.05 M sodium acetate buffer at pH 5.5 and an amount of enzyme was implemented for absorption measurement. The change of the absorption at 470 nm due to oxidation of guaiacol was recorded in 60 s-intervals for 3 min. One unit of peroxidase activity was defined as the enzyme quantity that increases O.D. 1.0 per min under standard conditions.

### Molecular weight estimation

The molecular weight of the protein was measured using Sephacryl S-200. In addition, SDS-PAGE with polyacrylamide gel (12%) and stacks (4%) was used to determine the purity and the subunit molecular weight of the purified enzyme. The Coomassie Blue staining method was used to detect the protein, and a prestained protein ladder (Thermo Scientific 26616) was used to determine the molecular mass^36^.

### Biochemical properties of *Z. jujuba* peroxidase

#### Effects of pH on the activity and stability of *Z. jujuba* peroxidase

*Z. jujuba* peroxidase activity was determined in the range of pH 4.0–9.0 using the following buffers: 50 mM sodium acetate (pH 4.0–6.0) and Tris–HCl (pH 6.5–9.0). To evaluate the pH stability, the residual activity after incubation for 24 hours at 4 and 28 °C was assessed at different pH values (pH 6.0–9.0)^37^.

#### Optimum temperature, activation energy and temperature quotient (Q_10_)

To determine the optimum temperature, the *Z. jujuba* peroxidase was examined at different temperatures between 25 and 80 °C. Standard assay conditions were applied during testing. The mixture was then cooled. The highest activity was reported as 100%^37^. The Arrhenius plot was used to determine the activation energy of the peroxidase (Ea). The effect of temperature on the reaction rate was demonstrated in terms of Q_10_, which is a variable that increases due to an increase in temperature of 10 °C^37^.

$${Q}_{10}=anti\,{\log }_{\varepsilon }\left(\frac{E\times 10}{R{T}^{2}}\right)$$

### Substrate specificity

The *Z. jujuba* peroxidase was evaluated for its preference for substrates, such as guaiacol, pyrogallol, 4-aminoantipyrine, ABTS, *O*-phenylenediamine and *O*-dianisidine. The enzyme activity was tested as outlined above.

### Kinetic constant (Km)

Km, Kcat and Vmax values of *Z. jujube* peroxidase were determined for guaiacol and H_2_O_2_ substrates. The Km and Vmax values were calculated from Lineweaver-Burk plots. Then, the catalytic efficiency value (Kcat/Km) was calculated for each substrate^38^.

### Effect of organic compounds on the Z. jujuba peroxidase activity

*Z. jujuba* peroxidase was incubated with several compounds (EDTA, isopropanol, β-mercaptoethanol, Urea, Triton x-100, NaN3 and SDS) for 15 min. the activity of the enzyme was measured as described above.

### Effect of metal ions

To investigate the effect of metals on *Z. jujuba* peroxidase activity, various metal ions, i.e., Fe^2+^, Ca^2+^, Cd^2+^, Ni^2+^, Cu^2+,^ Hg^2+^ and Zn^2+^ (final concentration 5.0 mM), were applied individually to tubes containing an assay-buffered substrate solution, and the mixtures were tested for enzyme activity under normal activity analysis.

### Thermostability Characteristics and Thermodynamic Parameters

The thermal stability profile was investigated by heating the purified enzyme within the temperature range of 55 to 70 °C, and residual activity was calculated from sterile aliquots withdrawn at periodic intervals using the following equation:

$${\rm{Residual}}\,{\rm{peroxidase}}\,{\rm{activity}}\,( \% )={{\rm{C}}}_{{\rm{t}}}/{{\rm{C}}}_{0}$$

where C_t_ and C_0_ describe the activities at time t (min) and time t = 0 min, respectively.

The enthalpy (ΔH*) was calculated using the relationship given in the following equation

$${\Delta {\rm{H}}}^{\ast }={{\rm{E}}}_{{\rm{a}}}^{\ast }-{\rm{RT}}$$

where R = 8.314 J K^−1^ mol^−1^ and is the universal gas constant and T indicates the absolute temperature (K).

The free energy of activation (ΔG*) at varying temperatures was determined from the relation shown in the following equation:

$${\Delta {\rm{G}}}^{\ast }=-\,{\rm{RT}}\,\mathrm{ln}\left(\frac{{\rm{Kdh}}}{{\rm{kT}}}\right)$$

where h is Planck’s constant (6.626 × 10^−34^ J·s), k is the Boltzmann constant (1.381 × 10^−23^ J·K^−1^), and the inactivation rate constant (K_d_) can be defined as the following equation:

$${{\rm{K}}}_{{\rm{d}}}=\left(\frac{KT}{h}\right){e}^{\left(\frac{{\Delta {\rm{H}}}^{\ast }}{{\rm{RT}}}\right){e}^{{(\Delta {\rm{S}}}^{\ast }/{\rm{R}})}}$$

Activation entropy (ΔS*) was calculated using the formula shown below:

$${\Delta {\rm{S}}}^{\ast }={(\Delta {\rm{H}}}^{\ast }-{\Delta {\rm{G}}}^{\ast })/{\rm{T}}$$

The enzyme half-life (t_1/2_) was described as the time after which the enzyme activity was decreased to one-half of the original activity and was calculated, as outlined by Gohel and Singh^39^, according to the following formula:

$${{\rm{t}}}_{1/2}=\,\mathrm{ln}\,2{/{\rm{K}}}_{{\rm{d}}}$$

The decimal reduction time or D-value, as stated by Pal and Khanum^40^, was described as the time of enzyme exposition at a given temperature, that preserves 10% of the resident operation.

$${\rm{D}}=2.303/{{\rm{K}}}_{{\rm{d}}}$$

The sensitivity factor (Z-value), which is described as the temperature increase required to reduce 90% of the D-value by one logarithmic cycle^40,41^, was determined from the plotted line curve of logD vs. T (°C).

## Results and discussion

**Figure 1:**
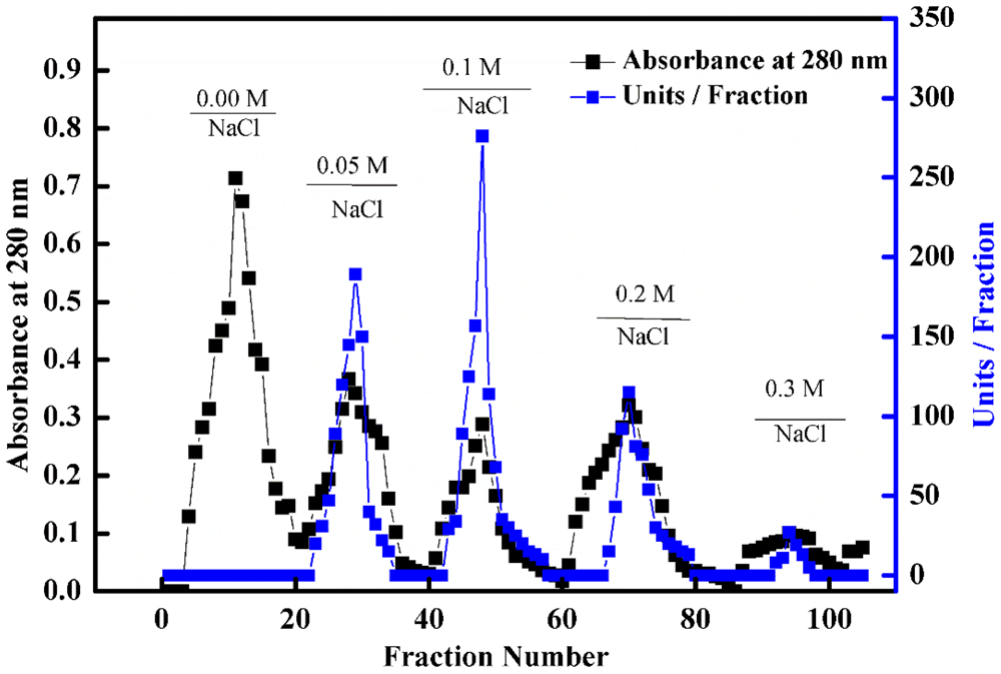
Chromatographic variations found during purification of *Z. jujuba* peroxidase. Profile of elution obtained from ion exchange chromatography on DEAE-Sepharose column previously equilibrated with 20 mM Tris-HCl buffer, pH 7.2 at a flow rate of 30 ml/h and 3 ml fractions.

**Figure 2:**
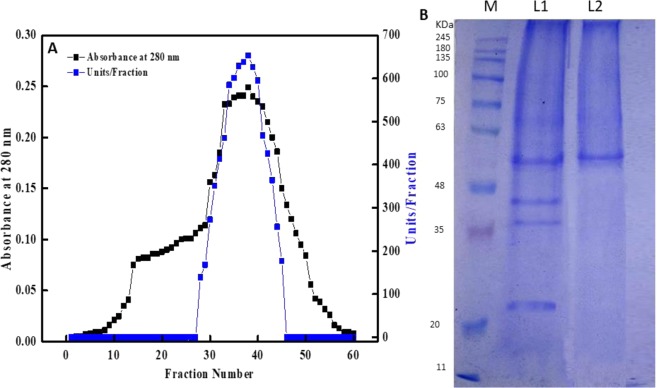
(**A**) Gel filtration profile on Sephacryl S-200 HR of DEAE-Sepharose fraction, the eluent volume of sample and marker proteins was collected (3 mL) at a flow rate of 30 mL/h. (**B**) SDS–PAGE of the purified peroxidase. Lane 1, low molecular weight protein markers, Lane 2, shows crude extract, Lane 3, shows purified *Z. jujuba* peroxidase.

**Figure 3:**
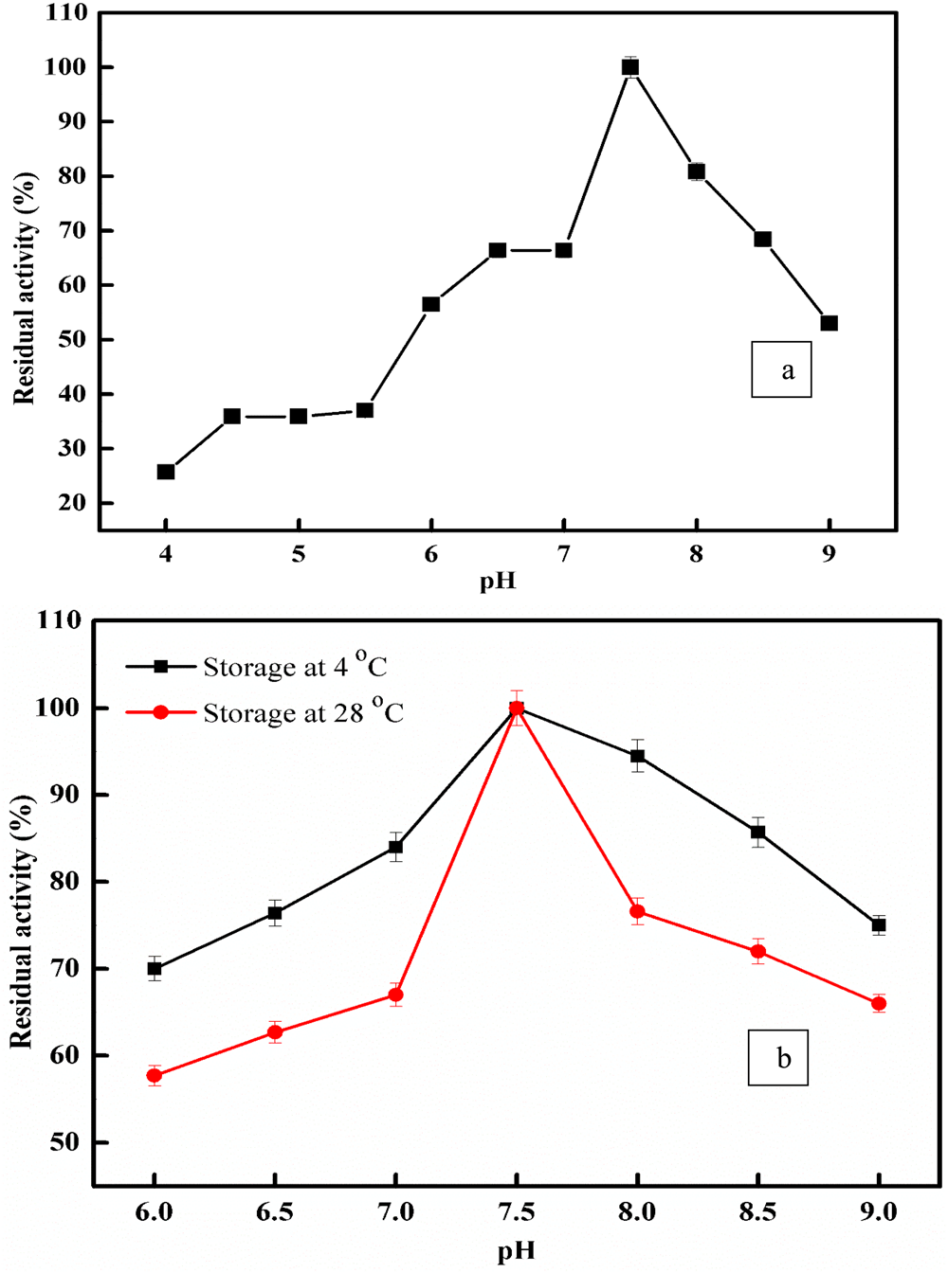
(**a**) pH optima and (**b**) pH stability of *Z. jujuba* peroxidase. One milliliter of a reaction mixture containing 0.008 M hydrogen peroxide, 0.04 M guaiacol, 0.05 M sodium acetate buffer (pH 4.0–6.0) and Tris–HCl (pH 6.5–9.0), and an amount of enzyme. The absorbance was recorded in 60 s-intervals for 3 min at 470 nm. Each point represents the mean of three experiments ± S.E.

peroxidase from *Ziziphus jujuba* was isolated and purified through the successive steps of ion exchange and gel filtration chromatography. The results for *Z. jujuba* peroxidase purification are summarized in Table 1. The fraction obtained by DEAE-Sepharose showed four peaks of peroxidase (POD) activity (Fig. 1). The fractions were collected from the elution profiles obtained with 0.05, 0.1, 0.2 and 0.3 M sodium chloride and designated as *Z. jujuba* peroxidases POD I, II, III and IV, respectively. The *Z. jujuba* peroxidase fraction POD II exhibited the highest activity and was separated on the Sephacryl S-200 column to obtain *Z. jujuba* peroxidase POD IIA (Fig. 2A), which exhibited the highest specific activity (7640 units/mg protein) along with an 18.9-fold enhancement of peroxidase purity and an overall recovery of 20%. SDS-PAGE can be implemented to obtain information on molecular weights and protein combinations^42^. The molecular weight and purity of the *Z. jujuba* peroxidase was investigated by Sephacryl S-200 chromatography and confirmed by SDS-PAGE (Figs. 2B and 1S). The gel filtration column was calibrated with different molecular weights (cytochrome C,12.4 kDa; carbonic anhydrase, 29 kDa; bovine albumin, 66 kDa; alcohol dehydrogenase,150 kDa; β-amylase, 200 kDa, and dextran blue, 2,000 kDa) (Figs. 2S and 3S). The crude POD had four bands with a major band at 56 kDa; after Sephacryl S-200 chromatography, a single band was detected for the purified *Z. jujube* peroxidase corresponding to a molecular weight of 56 kDa. Notably, the molecular masses of peroxidases from several plants were those of monomers, for example, the peroxidases of Arabian balsam (40 kDa), horseradish cv. Balady (56 kDa), broccoli (48 kDa), and palm leaf (48 kDa)^29,31,43–45^. The purified *Z. jujuba* peroxidase examined at distinct pH levels from pH 4 to 9, displayed optimum activity at pH 7.5 (Fig. 3a). The purified enzyme was robust under alkali pH values (the retained residual activity ranged from 81 to 53% in the pH range from 8 to 9). Other trials have revealed comparable outcomes where, in the pH range between 5 and 7.5, most peroxidases from various sources display optimal activity^29,45,46^. The enzyme lost nearly 75% of its activity at pH values lower than 4.0, while it maintained nearly 50% of its activity at pH 9.0. The pH influences the state of ionization of the side chain of amino acid enzymes. Loss of activity may be due to the haem-binding instability of the enzyme low pH values. Loss of activity may also derive from the denaturation of proteins or ionic shifts in the haem group at elevated pH values^47^. The impact of pH stabilization has also been examined in a wide pH range (6.0–9.0) for peroxidase activity. As illustrated in Fig. 3b, after 24 hours of incubation with pH values ranging from 6.0 to 9.0 (4 °C), the purified peroxidase retained almost 70% of its activity at pH levels above 7.0, with a maximum activity at pH 7.5, while the purified peroxidase maintained 66% of its initial activity at 28 °C.

**Table 1:** Purification scheme of *Z. jujuba* peroxidase. One milliliter of a reaction mixture containing 0.008 M hydrogen peroxide, 0.04 M guaiacol, 0.05 M sodium acetate buffer (pH 5.5), and an amount of enzyme. The absorbance was recorded in 60 s-intervals for 3 min at 470 nm.

Steps	T. units*	T. proteins mg	Specific activity (S.A) Unit/mg protein	Fold purification	Recovery 100%
Crude extract	5840	14.5	402.8	1	100
**Chromatography, DEAE-Sepharose (POD)**					
0.0 M NaCl	0.0	1.648	0.0	0.0	0.0
0.05 M NaCl (POD I)	1141	1.071	1065	2.6	19.5
**0.1 M NaCl** (POD 21)	**1723**	**0.329**	**5237**	**13**	**29.5**
0.2 M NaCl (POD 21)	256	0.116	1510	3.75	10.7
0.3 M NaCl (POD 21)	56	0.068	823	2	0.96
**Chromatography, Sephacryl S-200 (POD 21A)**					
(POD 21A)	**1169**	**0.153**	**7640**	**18.9**	**20**

*One unit of enzyme activity has been defined as the amount of enzyme that, under standard test conditions, increases the optical density by one U/min.

**Figure 4:**
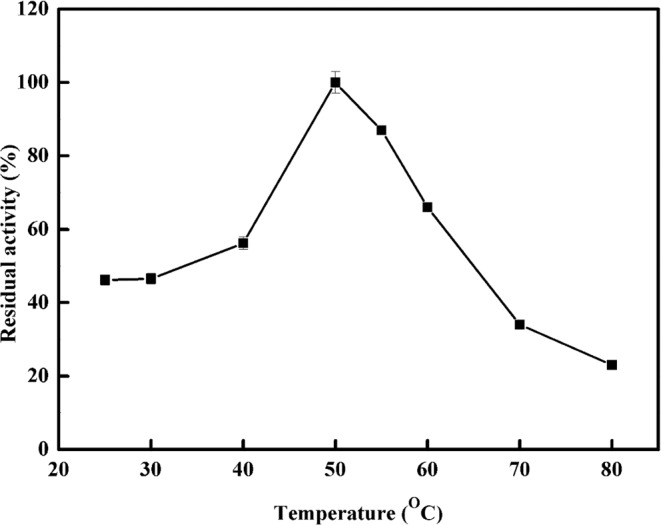
Optimum Temperature of *Z. jujuba* peroxidase. The enzyme activity was measured at various temperatures using the standard assay method. One milliliter of a reaction mixture containing 0.008 M hydrogen peroxide, 0.04 M guaiacol, 0.05 M sodium acetate buffer (pH 5.5), and an amount of enzyme. The absorbance was recorded in 60 s-intervals for 3 min at 470 nm. Each point represents the mean of three experiments ± S.E.

**Figure 5:**
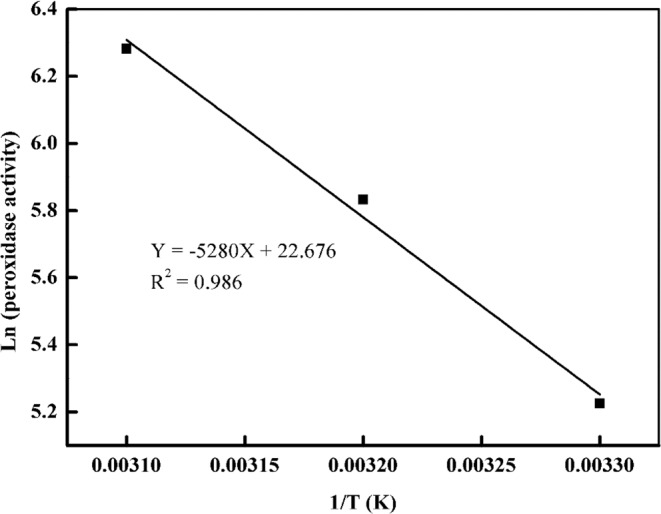
Determination of the activation energy based on Arrhenius plots. The enzyme activity was measured at various temperatures using the standard assay method. One milliliter of a reaction mixture containing 0.008 M hydrogen peroxide, 0.04 M guaiacol, 0.05 M sodium acetate buffer (pH 5.5), and an amount of enzyme.

The purified *Z. jujuba* peroxidase was investigated experimentally in order to determine the optimum temperature for its activity (Fig. 4). It was screened at different temperatures for this purpose. The obtained temperature profile showed that purified *Z. jujuba* peroxidase was highly active at 50 °C. The results are consistent with those of previous reports, where it was observed that the optimal temperature of the activity of peroxidase from various sources was observed in range between 40 and 65 °C^29,45,48^. The temperature sensitivity (Q_10_) and activation energy (E_a_*) are significant parameters that determine the enzyme stability and enzyme-substrate complex stability^49,50^. The activation energy of peroxidase was discovered to be 43.89 kJ mol^−1^ (Fig. 5), which is significantly higher than that found by McClaugherty (30 kJ mol^−1^)^51^. The temperature quotient for peroxidase was found to be 2.1, which is relatively greater than that reported for other peroxidases^51^.

**Figure 6:**
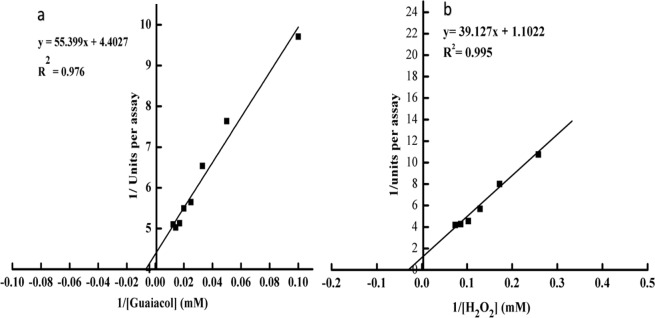
Lineweaver–Burk plot and Substrate saturation curve of *Z. jujuba* peroxidase activity in the presence of (**a**) guaiacol and (**b**) H_2_O_2_ concentrations as a fixed substrate. One milliliter of a reaction mixture containing 0.05 M sodium acetate buffer (pH 5.5), suitable amount of enzyme and concentrations of guaiacol ranging from 20 to 90 mM. and hydrogen peroxide ranging from 4 to 12 mM. The absorbance was recorded in 60 s-intervals for 3 min at 470 nm. Each point represents the mean of three experiments ± S.E.

**Figure 7:**
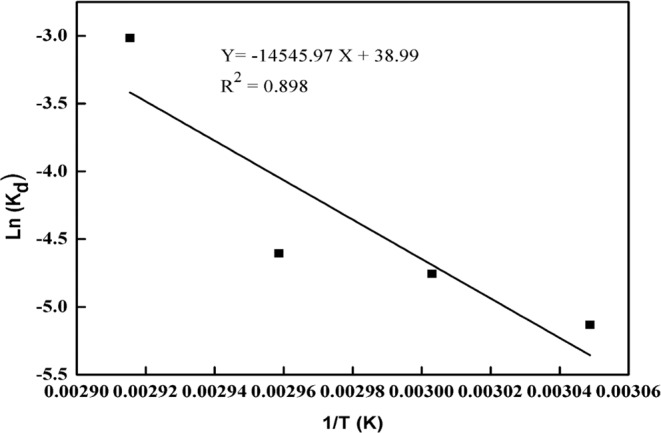
Determination of the deactivation energy depending on plots of Arrhenius. The enzyme was incubating at various temperatures, then the enzyme activity was measured using the standard assay method. One milliliter of a reaction mixture containing 0.008 M hydrogen peroxide, 0.04 M guaiacol, 0.05 M sodium acetate buffer (pH 5.5), and an amount of enzyme.

The Km and Vmax kinetic values of peroxidase determined by the Lineweaver–Burk plot for guaiacol and H_2_O_2_ substrate hydrolysis at 50 °C were 23.5 and 35.5 unit/ml and 0.23 and 0.91 mM, respectively (Fig. 6a,b), and the values of Kcat were 588 and 1538 s^−1^, respectively. The efficiency constant (Kcat/Km) was 25 and 43 for guaiacol and H_2_O_2_, respectively (Table 2), indicating high catalytic energy of the enzyme. The Km values of 46.5 and 4.81 mM for Guaiacol and H_2_O_2_, respectively were reported for peroxidase from Arabian balsam stems^29^, and 32.36 and 5.86 mM for guaiacol and H_2_O_2_, respectively, were reported for horseradish peroxidase^29^. A comparison with the above results indicates that *Z. jujuba* peroxidase has a greater activity for guaiacol than for H_2_O_2_. The inactivation rate constants and temperatures were significantly correlated. With the increasing temperature from 55 °C to 70 °C, the inactivation constant (K_d_) pace improved more than 8-fold. In addition, with increasing temperature, the half-life and decimal reduction time decreased as anticipated (Table 3). The *Z. jujuba* peroxidase showed good stability of a wide temperature range (55–65 °C), with a D-value of 388 to 230 min. However, the enzyme was found to be less stable at temperatures greater than 65 °C, as the D-values were discovered to be smaller. The deactivation energy E_a_^*^_(d)_ for *Z. jujuba* peroxidase calculated using an Arrhenius plot (Fig. 7) was found to be 120.93 kJ mol^−1^, which was significantly smaller than that of other reported enzymes^52^. The *Z. Jujuba* peroxidase had a Z value of 17.3, which suggests that the temperature must be increased by 17.3 °C to reduce 90% of the decimal reduction time. Thermodynamic properties (i.e., entropy, enthalpy, and the Gibbs free energy) are important parameters that provide precise proof of the unfolding of protein during inactivation^53^. Thermodynamic results for *Z. jujuba* peroxidase were calculated and are shown in Table 2. The ΔH* and ΔG* of the irreversible thermal inactivation of *Z. jujuba* peroxidase at 55 °C were equal to 118.20 and 83.42 kJ mol^−1^, respectively. The ΔH* and ΔG* were reduced to 118.08 and 81.33 kJ mol-1, respectively at 70 °C, while ΔS* increased to 107 jmol^−1^. Relatively low enzyme enthalpy values reflect its resistant nature, while increased values represent response to protein denaturation^54^. An increase in temperature caused the free energy to decrease, while entropy slightly increased, but the change in entropy was not significant. Low entropy values are noted to be exceptional in biological systems. With increasing temperature, the Gibbs free energy decreased, indicating that the enzyme had shown less resistance to heat unfolding at greater temperatures. The purified *Z. jujuba* peroxidase exhibited high activities against guaiacol (100%), *O*-phenylenediamine (87%), and *O*-dianisidine (67%). Moderate and low enzyme activities were obtained with 4-aminoantipyrine (37%), pyrogallol (21%) and ABTS (14%) (Table 4). This finding is similar to the patterns reported for horseradish peroxidase, which catalysed the oxidation of substrates in the order of guaiacol> *O*-phenylenediamine> *O*-dianisidine> 4-aminoantipyrine> pyrogallol> ABTS^55^. The peroxidase from *Ficus carica* latex had a specificity towards phenolic substrates in the order of guaiacol> *O*-phenylenediamine> *O*-dianisidine> pyrogallol> 4-aminoantipyrine^56^, while *Ficus sycomorus* latex peroxidase was found to follow the order of ABTS > *O*-phenylenediamine> guaiacol> 4-aminoantipyrine> *O*-dianisidine> pyrogallol^42^, and *Azadirachta indica* peroxidase had affinity towards phenolic substrates in the order of guaiacol> pyrogallol > *O*-dianisidine^57^. Ions are critical for the developmental activity of most plant peroxidases, and different metal ions influence peroxidase activity differently. Table 5 shows the influence of metal ions (5 mM) on the *Z. jujuba* peroxidase behaviour. It was observed that 5 mM Fe^2+^ and Ni^2+^ inhibited peroxidase activity by 4% and 44%, respectively, while 5 mM Zn^2+^, Cd^2+^ and Hg^2+^ inhibited peroxidase activity by 87%, 91% and 95%, respectively. On the other hand, the presence of 5 mM Cu^2+^ and Ca^2+^ could enhance the activity of *Z. jujuba* peroxidase by 21% and 8%, respectively. In the literature, a similarly slight inhibitory effect of Ni^2+^ on cotton peroxidases was reported^58^, while horseradish peroxidase was also reported to be inhibited by Zn^2+,^ Hg^2+^ and Cd^2+ ^^55^; additionally, the peroxidases isolated from *Ficus carica* latex were reported to be activated by Cu^2+ ^^56^. On the other hand, Ca^2+^ is reported to have an activity effect on citrus peroxidases^59^. The effect of various compounds on *Z. jujuba* peroxidase activity was determined, and the results highlight that this enzyme was slightly inhibited by isopropanol, EDTA, SDS, Triton X-100 and urea, strongly inhibited by NaN3 and completely inhibited by β-mercaptoethanol (Table 5). Sodium azide (NaN3) has been identified as an inhibitor for all peroxidases^60^ since it can interact with the metal ion of a metal enzyme which causes toxicity^61^; for instance, this chemical substance acts as an inhibitor of *Hevea brasiliensis* and *Jatropha curcasis* peroxidases^62,63^. *Z jujube* peroxidase was slightly inhibited by EDTA, a chelating agent, like those from *Hevea brasiliensis* and *Viscum angulatum*^62,64^. Furthermore, SDS, a good anionic detergent, has probably slightly inhibited the peroxidase activity due to a conformational change of the enzyme, which is in agreement with the results of a peroxidase from bitter gourd^65^. The *Z. jujuba* peroxidase retained 69% of its activity after treatment with 2 M urea. Similarly, the exposure of soluble HRP to 2 M urea resulted in a 60% retained activity^66^. In contrast, the *Z. jujuba* peroxidase exhibited 73% and 96% activity after exposure to 5% Triton X-100 and isopropanol, respectively. Similar results were obtained by Mohamed, *et al*.^66^.

**Table 2:** Kinetic parameters of *Z. jujuba* peroxidase.

Parameters	Guaiacol	H_2_O_2_
Km (mM)	23.47	35.5
Vmax µmole ml^−1^ min^−1^	0.23	0.91
Kcat (S^−1^)	588	1538
Kcat/Km	25	43

One milliliter of a reaction mixture containing 0.05 M sodium acetate buffer (pH 5.5), suitable amount of enzyme and concentrations of guaiacol ranging from 20 to 90 mM. and hydrogen peroxide ranging from 4 to 12 mM. The absorbance was recorded in 60 s-intervals for 3 min at 470 nm. Each point represents the mean of three experiments ± S.E.

**Table 3:** Thermodynamic parameters for irreversible inactivation of *Z. jujuba* peroxidase.

Temp. (K)	K_d_ (min^−1^)	t_1/2_ (min)	∆H* (kJ/mole)	ΔG* (kJ/mole	∆S* (J/mole)	D value	Ea*_(d)_ Kj/mole	Z value
328	0.0059	117.46	118.2	83.42	106	388	120.93	17.3
333	0.0086	80.58	118.16	83.387	104	268		
338	0.01	69.30	118.12	83.35	103	230		
343	0.0519	14.15	118.08	81.33	107	44		

The enzyme was incubating at various temperatures, then the enzyme activity was measured using the standard assay method. One milliliter of a reaction mixture containing 0.008 M hydrogen peroxide, 0.04 M guaiacol, 0.05 M sodium acetate buffer (pH 5.5), and an amount of enzyme.

**Table 4:** Substrate specificity of *Z. jujuba* peroxidase.

Substrate	*Z. jujube* peroxidase
Guaiacol	100 ± 0.011
O-Dianisidine	67 ± 0.075
4-Aminoantipyrine	37 ± 0.016
Pyrogallol	21 ± 0.049
ABTS	14 ± 0.010
O-Phenylenediamine	87 ± 0.022

One milliliter of a reaction mixture containing 0.008 M hydrogen peroxide, 0.04 M substrate, 0.05 M sodium acetate buffer (pH 5.5), and an amount of enzyme. The absorbance was recorded in 60 s-intervals for 3 min at 470 nm. Each point represents the mean of three experiments ± S.E.

**Table 5:** Effect of various metal ions and chemical compounds on the activity of the purified *Z. jujuba* peroxidase.

Metal ion and/or chemical compounds	Concentration	Relative activity %
control	—	100 ± 1.24
Cu^2+^	5 mM	121 ± 1.29
Fe^2+^		96 ± 1.42
Cd^2+^		9 ± 0.02
Ni^2+^		56 ± 0.18
Zn^2+^		13 ± 1.86
Hg^2+^		5 ± 0.03
Ca^2+^		108 ± 1.33
EDTA		89 ± 0.56
β-mercaptoethanol		0.0 ± 0.00
NaN3		27 ± 0.013
SDS		88 ± 0.41
Urea	2 M	69 ± 1.42
Triton X-100	5%	73 ± 0.45
Isopropanol	5%	96 ± 0.33

One milliliter of a reaction mixture containing 0.008 M hydrogen peroxide, 0.04 M guaiacol, 0.05 M sodium acetate buffer (pH 5.5), different concentration of metal ions and/or organic solvents and an amount of enzyme. The absorbance was recorded in 60 s-intervals for 3 min at 470 nm. Each point represents the mean of three experiments ± S.E.

## Conclusion

Peroxidase from *Z. jujuba* was purified and characterized. Purification via Sephacryl S-200 chromatography resulted in an 18.9-fold improvement of peroxidase activity with a 20% recovery. SDS-PAGE showed that the *Z. jujuba* peroxidase consisted of a single band with an apparent molecular weight of 56 kDa. Peroxidase from *Z. jujuba* is not temperature-sensitive and is very stable. The half-life was between 117.46 and 14.15 min at temperatures between 55 °C and 70 °C. The values of D (44.37–388 min), Z (17.3 °C), Q_10_ (2.1) and the high activation energy values suggest that a high amount of energy is required to initiate peroxidase denaturation. The optimum pH and temperature conditions for *Z. jujuba* peroxidase activity were a pH of 7.5 and 50 °C, respectively. The vegetables can, therefore, be blanched at 70 °C to inactivate *Z. Jujuba* peroxidase, prevent browning. Also the processing pH should be lower than pH 7 when the inhibition of peroxidase is required for food products from *Z. Jujuba*. Guaiacol is a natural phenolic product of chemical substances in plants (vegetables and fruits) which play an important role in enzymatic browning, as they constitute substrates for browning–enzymes. The guaiacol Km value was 23.5 unit/ml, which suggests that the enzyme has a high affinity for guaiacol. At the stage, the aim is to purifying and characterizing peroxidase and establish the best conditions that would give the best physical, biochemical and thermodynamic characteristics of the peroxidase enzyme. The optimum conditions obtained in the present study would serve as a basis to employ the peroxidase in the process of manufacturing and storage to improve the nutritional value and exterior quality.

## Supplementary information

Supplementary figures.
