# Supplementary material for: A novel peroxidase from Ziziphus jujuba fruit: purification, thermodynamics and biochemical characterization properties
Source: Sci Rep. 2020 May 14;10:8007. doi: 10.1038/s41598-020-64599-9 (PMC7224213; doi:10.1038/s41598-020-64599-9)
Supplement: Supplementary file 1 — Supplementary figures. [file 41598_2020_64599_MOESM1_ESM.pdf]

A novel peroxidase from *Ziziphus jujuba* fruit: purification, thermodynamics and biochemical characterization properties

Mustafa Zeyadi[1], Yaaser Q. Almulaiky[2,3*]

[1]Department of Biochemistry, Faculty of Science, King Abdulaziz University, Jeddah, P. O. Box 80200, Jeddah 21589, Saudi Arabia

[2]University of Jeddah, College of Sciences and Arts at Khulais, Department of Chemistry, Jeddah, Saudi Arabia

[3]Chemistry Department, Faculty of Applied Science, Taiz University, Taiz, Yemen

*Correspondence to: Dr. Yaaser Q. Almulaiky

Tel: +966566880264

E-mail: yaseralmoliki@hotmail.com

Molecular weight estimation

SDS-PAGE with polyacrylamide gel (12%) and stacks (4%) was used to determine the purity and the subunit molecular weight of the purified enzyme.

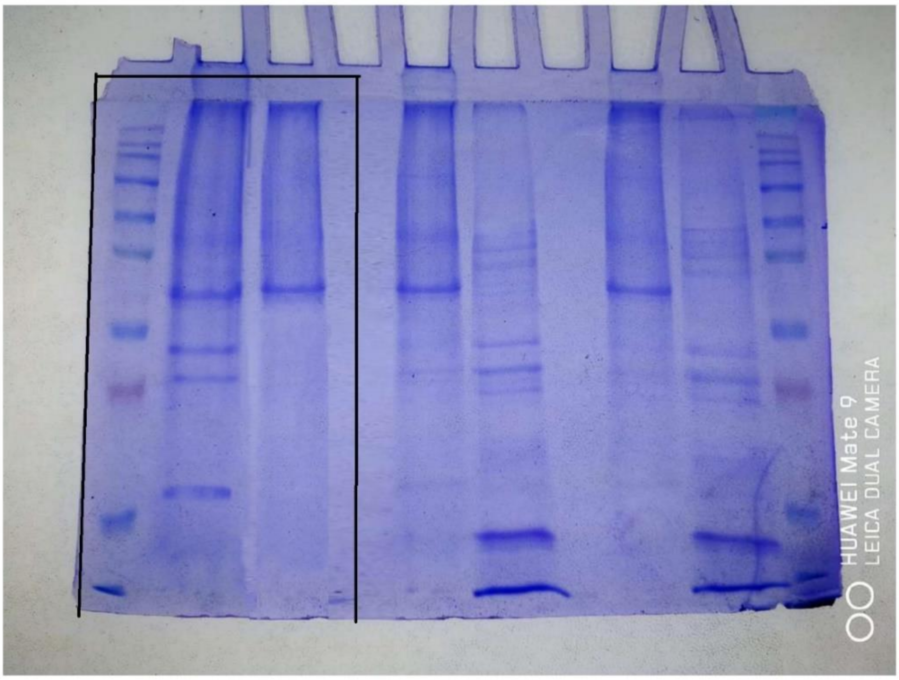

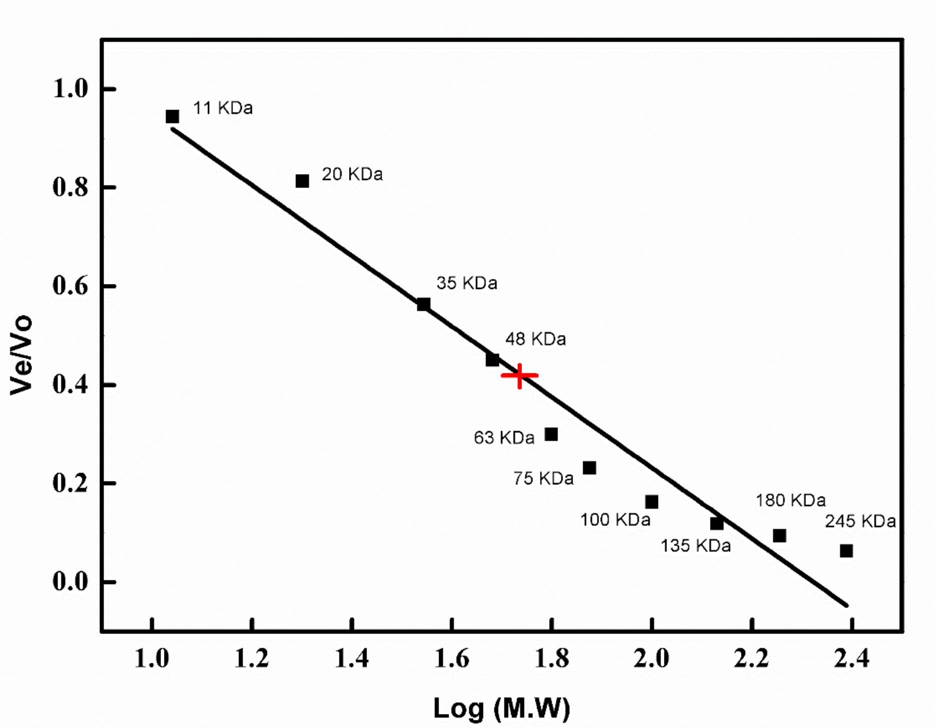

Figure 1S SDS Supplementary Information

Molecular weight determination by gel filtration

Molecular weight was determined by gel filtration technique using a Sephacryl S-200. The column was calibrated with cytochrome C (12.4 kDa), carbonic anhydrase (29 kDa), bovine albumin (66 kDa), alcohol dehydrogenase (150 kDa), β-amylase (200 kDa) (Figures 2S). Dextran blue (2,000 kDa) was used to determine the void volume (VO) (Figure 3.6). Protein solutions were applied to the same column and developed using the same equilibration buffer at a flow rate of 30 ml/h, and fractions of 3 ml volume were collected. A calibration curve was constructed by plotting log molecular weight versus V_e/ V_O, where V_e was the elution volume and V_O was the void volume (Figure 3S).

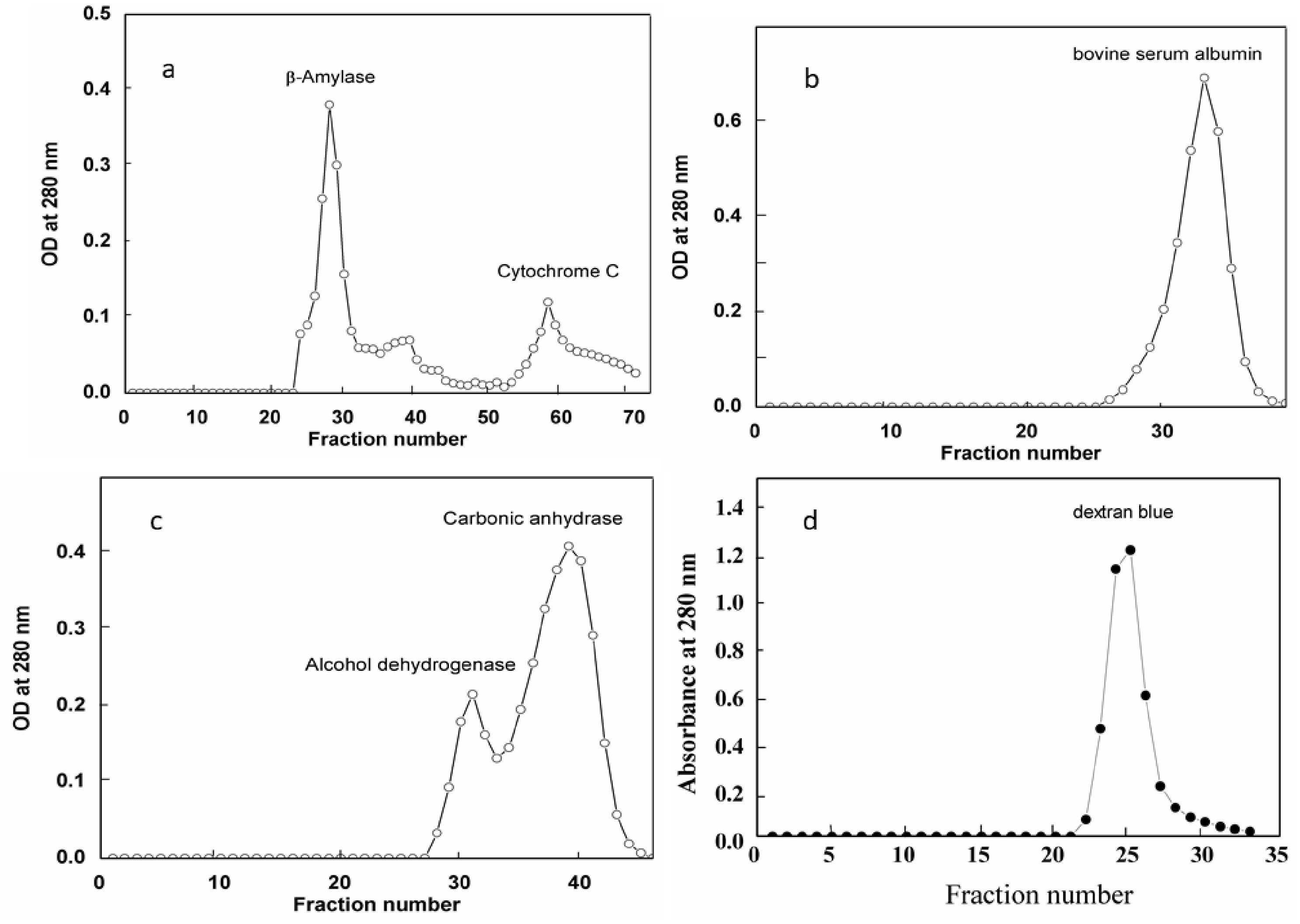

Figure 2S Gel filtration of β-amylase and cytochrome C(a), bovine serum albumin (b), alcohol dehydrogenase and carbonic anhydrase (c), and dextran blue (d) on Sephacryl S-200 column.

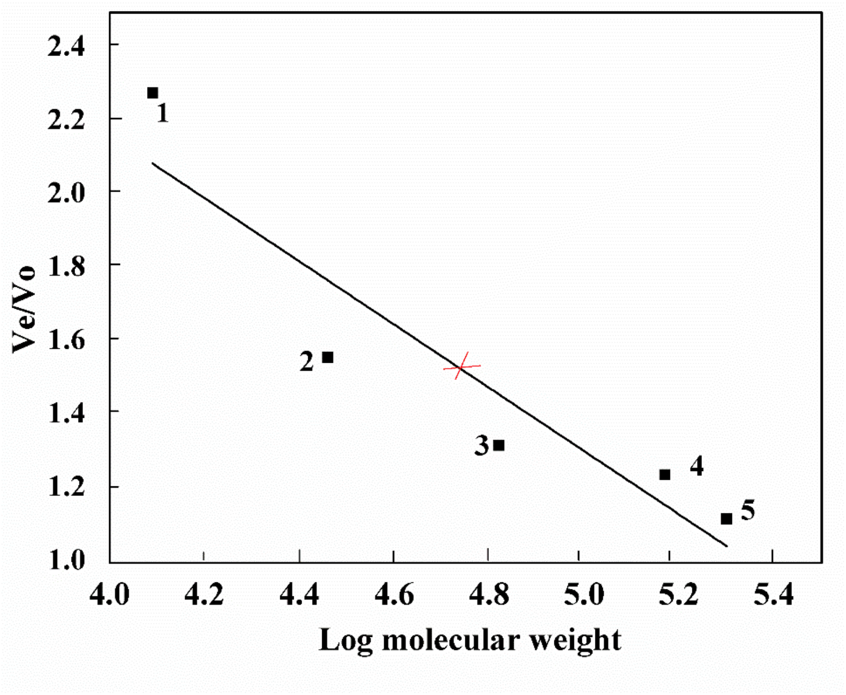

Figure 3S Calibration curve for estimation of the molecular weight by gel filtration on Sephacryl S-200 column: 1) Cytochrome C (12.4 kDa); 2) Carbonic anhydrase (29 kDa); 3) Bovine albumin (66 kDa); 4) Alcohol dehydrogenase (150 kDa); 5) β-Amylase (200 kDa). Void volume was determined with Dextran blue (2,000 kDa). The eluent volume of sample and marker proteins was collected (3 mL) at a flow rate of 30 mL/h.
